# Kynurenic Acid Analog Attenuates the Production of Tumor Necrosis Factor-α, Calgranulins (S100A 8/9 and S100A 12), and the Secretion of HNP1–3, and Stimulates the Production of Tumor Necrosis Factor-Stimulated Gene-6 (TSG-6) but Does Not Alter IL-17 Levels in Whole-Blood Cultures of Patients with Spondyloarthritis

**DOI:** 10.3390/ijms262411801

**Published:** 2025-12-06

**Authors:** Borisz Varga, Gergely Toldi, László Vécsei, Yvette Mándi, Attila Balog

**Affiliations:** 1 Department of Rheumatology and Immunology, University of Szeged, 6725 Szeged, Hungary; varga.borisz@med.u-szeged.hu; 2 Liggins Institute, University of Auckland, Auckland 1023, New Zealand; gergely.toldi@auckland.ac.nz; 3 Department of Neurology, University of Szeged, 6725 Szeged, Hungary; vecsei.laszlo@med.u-szeged.hu; 4 Department of Medical Microbiology, University of Szeged, 6720 Szeged, Hungary; mandi.yvette@med.u-szeged.hu

**Keywords:** kynurenine, kynurenic acid, spondyloarthritis, TSG-6, TNFα-stimulated gene-6, calgranulins, HNP1–3, alpfa-defensin, interleukine-17

## Abstract

Kynurenic acid (KYNA) has recognized anti-inflammatory and immunosuppressive properties. Previous studies demonstrated that KYNA reduces TNF-α, S100A12, S100A8/9, and α-defensin production while increasing tumor necrosis factor-stimulated gene-6 protein (TSG-6) levels in rheumatoid arthritis. This study evaluated a synthetic KYNA analog’s effects on TNF-α, S100A8/9, S100A12, α-defensin, and interleukin-17 (IL-17) production and TSG-6 expression in ankylosing spondylitis (AS) and psoriatic arthritis (PsA). Peripheral blood mononuclear cells from 54 AS and 38 PsA patients and 11 healthy controls were stimulated with heat-inactivated *Staphylococcus aureus* (SA1). Parallel cultures were pretreated with the KYNA analog. Cytokine and alarmin concentrations were measured by ELISA. SA1 stimulation increased TNF-α, TSG-6, calprotectin, and α-defensin production, with minimal effects on S100A12 and none on IL-17. The KYNA analog significantly reduced SA1-induced TNF-α, calprotectin, and α-defensin levels and enhanced TSG-6 production, without affecting S100A12 or IL-17. Notably, the TNF-α inhibitory and TSG-6 stimulatory effects were inversely correlated. Conclusion: KYNA analogs may exert anti-inflammatory effects via TSG-6 upregulation, contributing to the suppression of key cytokines. These findings support further exploration of KYNA derivatives as therapeutic options in immune-mediated diseases including AS and PsA.

## 1. Introduction

Spondyloarthropathies (SpA) form a diverse group of chronic inflammatory diseases with both autoimmune and autoinflammatory components. Among them, ankylosing spondylitis (AS) and psoriatic arthritis (PsA) are the most extensively studied diseases. AS typically involves axial joints (axSpA), while PsA presents with peripheral arthritis (pSpA), although clinical features often overlap [1]. The pathogenesis of SpA is multifactorial, involving genetic predisposition (e.g., human leukocyte antigen (HLA) B27), epigenetic modifications, and environmental factors such as mechanical microtrauma and intestinal dysbiosis) [2]. These contribute to an abnormal immune response with the overproduction of proinflammatory cytokines—primarily tumor necrosis factor-α (TNF-α), interleukin (IL)-17, and IL-23—leading to joint damage and extra-articular manifestations [3].

In recent years, attention has turned toward the tryptophan–kynurenine metabolic pathway as a potential contributor to SpA pathogenesis [4]. Kynurenic acid (KYNA), a neuroactive metabolite produced via tryptophan degradation by the enzymes tryptophan 2,3-dioxygenase (TDO) and indoleamine 2,3-dioxygenase (IDO), has emerged as a key regulator of both neuroimmune and inflammatory processes. IDO activity is upregulated under inflammatory conditions, particularly in response to interferon-γ (IFN-γ), leading to increased production of KYNA and related metabolites. KYNA acts as an endogenous antagonist of glutamatergic NMDA, AMPA, and kainate receptors, as well as the α7 nicotinic acetylcholine receptor. These actions confer neuroprotective effects, especially under excitotoxic conditions [5]. In addition, KYNA exerts anti-inflammatory effects through the activation of G-protein-coupled receptor 35 (GPR35) [6] and the aryl hydrocarbon receptor (AhR) [7], both of which play roles in modulating innate and adaptive immunity. Reduced KYNA levels have been implicated in neurodegenerative diseases, while elevated levels are associated with schizophrenia, underscoring its diverse biological functions [5].

Immunologically, KYNA has been shown to suppress effector T-cell responses and promote the differentiation of regulatory T-cells, shifting the Th1/Th2 balance toward an anti-inflammatory Th2 phenotype. These effects are largely mediated via AhR signaling. In previous studies, KYNA and its synthetic analogs significantly inhibited TNF-α production in human peripheral blood mononuclear cells and in the U-937 monocyte cell line. Simultaneously, we observed upregulation of TNF-stimulated gene-6 (TSG-6), a 35 kDa hyaluronan-binding protein with well-established anti-inflammatory and tissue-protective roles [8,9]. TSG-6 modulates inflammatory signaling via Toll-like receptor (TLR) inhibition, particularly through interaction with the CD44 receptor, leading to reduced production of TNF-α and IL-6. It also contributes to the induction of regulatory T-cells and M2 macrophages, as well as the suppression of neutrophil extracellular trap formation and reactive oxygen species release [10]. TSG-6 expression is closely linked to KYNA activity via AhR-dependent mechanisms, and we confirmed that KYNA analogs can significantly elevate TSG-6 levels in both cell lines and primary human monocytes [8,9].

In our previous studies, SZR-72 was synthesized by direct amidation of KYNA and its potential effects both in neurologic and immunologic diseases were investigated. The results of our previous studies suggest that the receptor affinity, metabolic stability, and neuroactive effects of the new analog SZR72 are comparable with those of KYNA. Furthermore, in contrast with KYNA, it crosses the blood–brain barrier [11]. Additionally, our results also confirmed that SZR72 has a more potent immunoregulatory effect than KynA on human mononuclear cells, monocytes, and granulocytes [12]. The immunmodulatory effect of SZR72 was also described in vitro using whole-blood samples from patients with rheumatoid arthritis in our recent study [9]. The new analog SZR72 can therefore be considered a promising candidate for clinical trials, including neurological and immunological studies [9].

SZR72 significantly reduced levels of key inflammatory mediators including TNF-α, human neutrophil peptide 1–3 (HNP1–3 or α-defensin), and alarmins such as S100A8/9 (calprotectin) and S100A12 (also known as extracellular newly identified receptor for advanced glycation end-products-binding protein—EN-RAGE). These proteins are considered damage-associated molecular patterns (DAMPs), produced primarily by activated neutrophils and monocytes in response to inflammation. They exert their effects via the engagement of TLR4 [13] and RAGE receptors [14,15], resulting in nuclear factor kappa-light-chain-enhancer of activated B cells (NF-κB) activation and perpetuation of inflammation. Both S100 proteins are recognized markers of disease activity in autoimmune and chronic inflammatory conditions [14,16,17].

Alpha-defensins are antimicrobial peptides that contribute to innate immune defense and the propagation of inflammation through enhanced phagocytosis and cytokine production [18,19]. SZR72 treatment was found to decrease these inflammatory mediators while simultaneously increasing TSG-6 levels, indicating a broad anti-inflammatory effect across multiple immune pathways [8,9].

Direct measurement of IL-17 and IL-23 in serum is challenging due to their low circulating concentrations [20]. In contrast, IL-17-induced proteins such as α-defensins and alarmins are present at higher, more measurable levels, making them reliable surrogate markers for IL-17 pathway activity. Notably, calprotectin and related molecules have been implicated in SpA pathogenesis and are detectable in significant concentrations in serum, reinforcing their diagnostic and prognostic utility [20].

Taken together, our findings suggest that KYNA and its synthetic derivatives, particularly SZR72, exert significant immunomodulatory effects by inhibiting key proinflammatory cytokines and innate immune activators while promoting anti-inflammatory mediators such as TSG-6 [9]. These results support the further exploration of KYNA-based compounds as potential therapeutic agents in SpA and related autoimmune diseases. Our current study aims to assess the impact of SZR72 on the production of TNF-α, IL-17, HNP1–3, S100A8/9, S100A12, and TSG-6 in the peripheral blood of SpA patients, with the goal of clarifying its mechanism of action and therapeutic potential.

## 2. Results

We first compared TNF-a, TSG-6, calprotectin, HNP-1-3, S100A12, and IL-17 levels in subgroups of SpA patients divided according to sex (male, female), therapy (nonsteroidal anti-inflammatory drug [NSAID], conventional synthetic disease-modifying antirheumatic drug [csDMARD] only, csDMARD and biologic DMARD [bDMARD]), disease activity (mild, moderate, severe), HLA-B27 positivity, C-reactive protein (CRP) positivity, and peripheral or axial articular involvement. The following comparisons showed statistically significant differences.

### 2.1. KYNA Analog SZR72 Attenuates TNF-α Production in the Human Whole-Blood Cells of Healthy Controls and of Patients with SpA Stimulated by Heat-Inactivated Staphylococcus aureus

In our earlier work, we investigated the impact of the KYNA analog SZR72 on TNF-α production in blood samples from healthy donors and patients with RA. Building on these findings, in the present study we applied SZR72 at a concentration of 500 μM, which had previously been identified as the most effective dose. In heat-inactivated *S. aureus* (SA1)-stimulated blood cultures from healthy controls, TNF-α levels increased markedly, ranging from 140 to 1030.6 pg/mL (median: 444.2 pg/mL), compared with basal control values of 7.8–350 pg/mL (median: 8.9 pg/mL). Treatment with SZR72 significantly reduced TNF-α production, lowering the median level to 280 pg/mL. To assess whether this effect varied according to clinical disease status, patients with SpA were stratified into subgroups. Across all groups, SZR72 consistently suppressed SA1-induced TNF-α production, with no significant subgroup-specific differences. In patients with remission or mild disease, basal TNF-α levels (median: 72.8 pg/mL) rose to 280 pg/mL after SA1 stimulation, but SZR72 treatment significantly reduced this response to 131 pg/mL. In the moderate disease group, median basal concentrations were 79 pg/mL, increasing to 333 pg/mL following stimulation; this induction was again significantly attenuated by SZR72, lowering levels to 145 pg/mL. Interestingly, in patients with severe disease, basal TNF-α concentrations were comparatively lower (median: 42.9 pg/mL). After SA1 stimulation, levels rose to 339 pg/mL, but SZR72 significantly reduced this increase, resulting in a median of 199 pg/mL (Table 1).

### 2.2. The Effect of KYNA Analog SZR72 on TSG-6 Production in Human Whole-Blood Cells of Healthy Controls and of Patients with SpA Stimulated by Heat-Inactivated Staphylococcus aureus

In healthy controls, TSG-6 levels in basal blood cultures increased following SA1 stimulation and were further elevated by the addition of 500 µM SZR72. At this concentration, SZR72 significantly enhanced TSG-6 production in SA1-induced samples (median 13.6 ng/mL vs. 9.22 ng/mL). These findings are in line with our earlier observations showing that KYNA and its analogs upregulate TSG-6 expression at both the RNA and protein levels [8,9]. We then examined whether SZR72 exerted differential effects among patient subgroups. In all groups, SZR72 increased TSG-6 production in SA1-induced cultures, although the magnitude of this effect varied with disease activity. Patients with severe disease exhibited the lowest TSG-6 concentrations (median basal 0.8 ng/mL; SA1-induced 4.3 ng/mL; SZR72-treated 6.9 ng/mL). In contrast, healthy controls showed the highest values (median basal 5 ng/mL; SA1-induced 9.2 ng/mL; SZR72-treated 13.6 ng/mL) (Table 1).

### 2.3. The Effect of KYNA Analog SZR72 on Calprotectin Production in the Human Whole-Blood Cells of Healthy Controls and of Patients with SpA Stimulated by Heat-Inactivated Staphylococcus aureus

The role of calprotectin as a biomarker in SpA has gained considerable attention in recent years [13,16,20,21]. To further explore this, we examined the effect of the KYNA analog SZR72 on calprotectin production in whole-blood cultures. Basal calprotectin levels ranged from 390 to 1902 ng/mL (median 824 ng/mL), and SA1 stimulation induced a marked increase to 4726–15,500 ng/mL (median 12,350 ng/mL). Treatment with 500 µM SZR72 significantly reduced SA1-induced calprotectin levels to a median of 5892 ng/mL (Table 1). We next assessed whether disease activity influenced this response. In all patient subgroups, SZR72 suppressed SA1-mediated calprotectin production, though the magnitude varied with disease severity. Patients in remission/with mild disease exhibited the lowest levels (median basal 849 ng/mL; SA1-induced 8525.9 ng/mL; SZR72-treated 5806 ng/mL). In contrast, patients with severe disease showed higher basal calprotectin concentrations (median 3733 ng/mL), which increased to 25,950 ng/mL upon SA1 induction but were significantly reduced by SZR72 treatment to 22,250 ng/mL (Table 1).

### 2.4. The Effect of the KYNA Analog SZR72 on S100A12 (EN-RAGE) Production in the Human Whole-Blood Cells of Normal Controls and of Patients with SpA Stimulated by Heat-Inactivated Staphylococcus aureus

Since S100A12 (EN-RAGE) is predominantly produced by granulocytes, its regulation may provide important insights into granulocyte-driven inflammation in SpA. Therefore, we assessed the effect of the KYNA analog SZR72 on EN-RAGE production in whole-blood cultures. In control individuals, basal EN-RAGE concentrations ranged from 1150 to 12,000 ng/mL, with a median of 8306 ng/mL. Following SA1 stimulation, levels increased to 10,074–16,955 ng/mL (median 13,098 ng/mL). Treatment with 500 µM SZR72 modestly reduced SA1-induced EN-RAGE production to a median of 12,932 ng/mL; however, this reduction did not reach statistical significance. To further explore potential disease-related differences, patients were stratified according to disease activity. In the remission/mild group, basal EN-RAGE levels had a median of 11,864 ng/mL, which increased to 13,659 ng/mL after SA1 induction and decreased to 12,440 ng/mL with SZR72 treatment. In the moderate group, the median basal level was 12,500 ng/mL, which rose to 14,416 ng/mL upon SA1 stimulation and declined to 13,136 ng/mL following SZR72 exposure. Patients with severe disease displayed the highest basal EN-RAGE levels (median 19,288 ng/mL), which showed only a slight increase after SA1 stimulation (19,678 ng/mL) and a minor, non-significant decrease following SZR72 treatment (19,078 ng/mL) (Table 1).

### 2.5. The Effect of the KYNA Analog SZR72 on HNP1–3 Production in the Human Whole-Blood Cells of Normal Controls and of Patients with SpA Stimulated by Heat-Inactivated Staphylococcus aureus

Incubation of whole blood from healthy donors with SA1 led to a marked increase in HNP1–3 secretion. The basal median concentration was 121 ng/mL, which rose to 540 ng/mL following SA1 stimulation. Pretreatment with 500 µM SZR72 significantly inhibited this response, reducing HNP1–3 levels to a median of 387 ng/mL. In patients with SpA in remission or with mild disease, basal HNP1–3 levels were slightly lower (median 98 ng/mL) but increased to 520 ng/mL after SA1 induction. SZR72 treatment significantly suppressed this induction, reducing HNP1–3 concentrations to 280 ng/mL. In the moderate disease group, median basal levels were 119 ng/mL, which increased to 535 ng/mL after SA1 stimulation. SZR72 again significantly inhibited this elevation, lowering HNP1–3 levels to 272 ng/mL (statistically confirmed by Friedman’s test and Dunn’s post-test). Patients with severe disease exhibited strikingly higher basal HNP1–3 concentrations (median 1828 ng/mL), likely reflecting enhanced neutrophil degranulation. Following SA1 stimulation, levels further increased to 6345 ng/mL. Treatment with SZR72 significantly reduced this response, bringing HNP1–3 concentrations down to 4850 ng/mL (Table 1).

### 2.6. The Effect of the KYNA Analog SZR72 on IL-17 Production in the Human Whole-Blood Cells of Normal Controls and of Patients with SpA Stimulated by Heat-Inactivated Staphylococcus aureus

IL-17 is a central cytokine in the pathogenesis of SpA, and therefore we assessed its production under the same experimental conditions. In both healthy controls and patients with SpA, IL-17 concentrations in whole-blood cultures showed marked variability, ranging from undetectable to very high levels. Importantly, no consistent changes were observed in response to SA1 stimulation or SZR72 treatment. Likewise, the stratification of patients according to disease activity, therapy, or clinical phenotype did not reveal significant differences. These findings suggest that IL-17 production in ex vivo whole-blood cultures is highly heterogeneous and not reliably modulated by SA1 stimulation or SZR72 under the applied experimental conditions.

## 3. Discussion

Several recent papers supported the significant role of tryptophan metabolites in the modulation of immune cells [22] and the kinurine pathway in the development of immune-related diseases [23]. An integrated cytokine and kynurenine network was also described in neuroimmune communication [24]. In our work, we focused on the cytokine and kynurine network in inflammatory rheumatic diseases such as AS and PsA.

Kynurenic acid (KynA), a broad-spectrum antagonist of excitatory amino acid receptors, may serve as a protective agent in neurological disorders and inflammatory rheumatic diseases as well. In our previous experiments, the inhibitory effects of kynurenic acid and a kynurenic acid analog, SZR72, on TNF-α production by mononuclear cells and HNP1–3 secretion by neutrophils were confirmed [12]. Furthermore, our results suggested that the new KynA analog SZR72 has a more potent immunoregulatory effect than KynA on human mononuclear cells, monocytes, and granulocytes. We also justified the immunoregulatory effect of SZR72 in whole-blood cultures of patients with rheumatoid arthritis (RA). SZR72 attenuated the production of tumor necrosis factor-α, calgranulins (S100A 8/9 and S100A 12), and the secretion of HNP1–3, and stimulated the production of tumor necrosis factor-stimulated gene-6 in RA [9]. The precise role of the kinurenic acid pathway and the potential immunomodulatory effect of KynA analogs in SpA has not been investigated. We hypothesized similar changes in SpA as in RA.

In this study, we investigated the in vitro production of TNF-a, TSG-6, and the calgranulins as calprotectin and EN-RAGE, together with HNP1–3 in spondyloarthropathic patients stratified by clinical and therapeutic parameters. We also assessed the immunomodulatory effects of the KYNA analog SZR72 following SA1 stimulation in vitro in the peripheral blood of patients with SpA and PsA.

SA1 stimulation is a conventional model for the induction of cytokine production. In our previous works using in vitro cell lines, human monuclear cells and whole-blood samples from patients with rheumatoid arthritis SA1 stimulation was also used as a cytokine inducer. We also used this SA1 as cytokine inducer in SpA independently from the defined inflammatory rheumatic disease [25].

**Figure 1:**
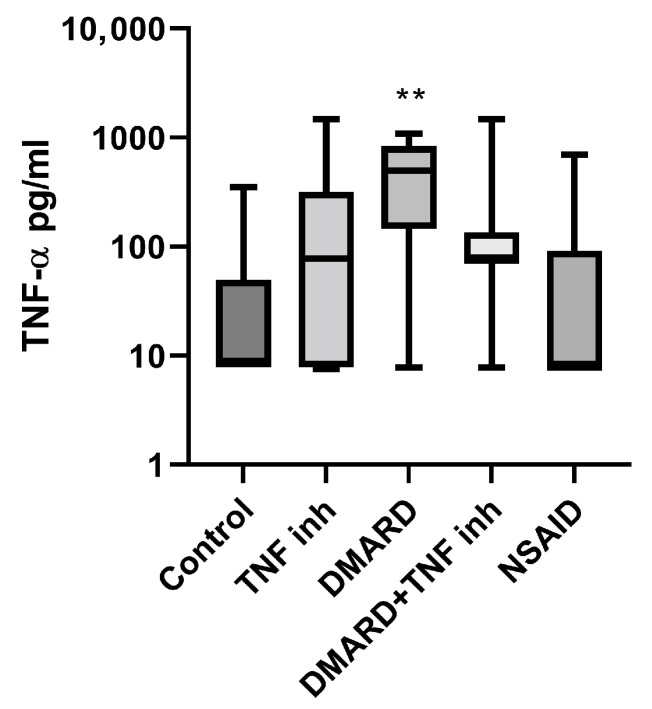
TNF-a levels in supernatants of whole-blood cultures of healthy controls and patients on various therapeutic regimes. ** *p* < 0.01 vs. Control.

Among the proinflammatory markers examined, TNF-α plays a well-established central role in the pathogenesis of AS and PsA. In line with previous studies, we observed elevated TNF-α levels in patients on csDMARD therapy compared to healthy controls and other therapeutic regimes (Figure 1). Although csDMARDs have proven efficacy in peripheral joint inflammation, they exhibit limited direct TNF-inhibitory effects compared to bDMARDs, which may explain the observed elevation. These findings underscore the differential impact of conventional vs. targeted immunomodulatory therapies on TNF-α regulation. The low TNF-α concentration in the patient group treated with only NSAID can be explained by the low overall disease activity in this group.

Interestingly, TSG-6 was significantly higher in healthy controls compared to patients with severe disease activity and also in CRP-negative patients compared to CRP-positive patients, supporting its compensatory role in counterbalancing low-grade inflammation. TSG-6 exerts immunosuppressive effects and its expression often forms part of a negative feedback loop aimed at limiting excessive inflammation [26], meaning that TSG-6 levels can be relatively higher during moderate or mild disease activity, when endogenous anti-inflammatory responses are still active. In contrast, during highly active inflammation, this regulatory system may become overwhelmed or exhausted, leading to comparatively lower TSG-6 levels despite more severe disease. These findings are consistent with our previous study, where we observed that TSG-6 concentrations were higher in patients with lower disease activity and in healthy controls compared to those with more severe RA [9]. However, further investigation is warranted, as other reports have demonstrated the opposite trend, with serum TSG-6 levels showing a positive correlation with disease activity in RA [27] and in murine arthritis models [28].

**Figure 2:**
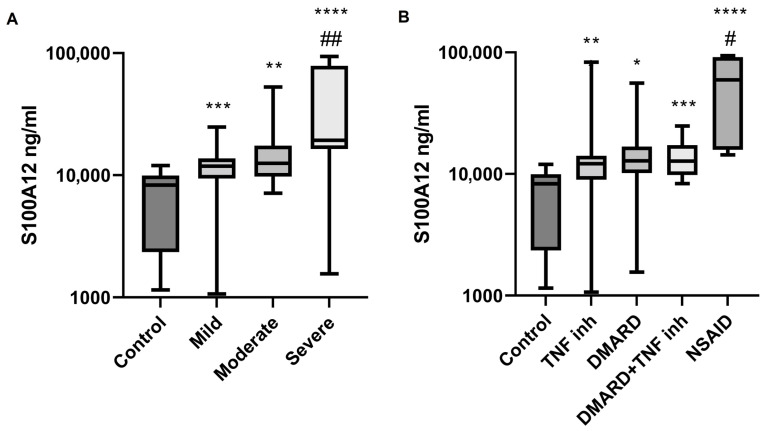
(**A**): S100A 12 levels in supernatants of whole-blood cultures of healthy controls and patients of various disease severities. ** *p* < 0.01 vs. Control, *** *p* < 0.001 vs. Control, **** *p* < 0.0001 vs. Control, and ## *p* < 0.01 vs. Mild. (**B**): S100A 12 levels in supernatants of whole-blood cultures of healthy controls and patients on various therapeutic regimes. * *p* < 0.05 vs. Control, ** *p* < 0.01 vs. Control, *** *p* < 0.001 vs. Control, **** *p* < 0.0001 vs. Control, and # *p* < 0.05 vs. Mild.

**Figure 3:**
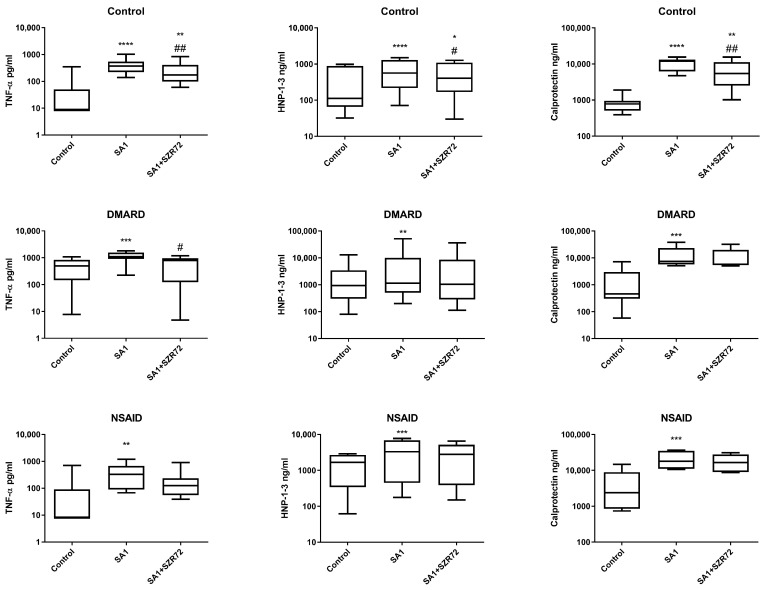
TNF-α, HNP-1-3 and calprotectin levels in supernatants of whole-blood cultures of healthy controls, patients on DMARD therapy and patients on NSAID therapy. * *p* < 0.05 vs. Control, ** *p* < 0.01 vs. Control, *** *p* < 0.001 vs. Control, **** *p* < 0.0001 vs. Control, # *p* < 0.05 vs. SA1, ## *p* < 0.01 vs. SA1.

Calprotectin (S100A8/9) is increasingly recognized as a robust indicator of disease activity in SpA [29]. In our study, we observed a clear positive correlation with disease severity, as calprotectin levels were significantly higher in patients with severe disease and in CRP-positive patients compared to CRP-negative patients (Figure 2). Similarly, HNP-1–3 (α-defensin) levels were also markedly increased in patients with severe disease, both markers correlating strongly with systemic inflammation. Since calprotectin and HNP-1–3 are secreted by activated neutrophils and monocytes, their elevation appears to reflect the intensity of innate immune activation. The strong association of both biomarkers with higher disease severity supports their potential clinical utility in monitoring disease activity [21]. Notably, while patients receiving NSAID, DMARD, or TNF inhibitor monotherapy exhibited elevated HNP-1–3 levels, those on combined DMARD and TNF inhibitor therapy did not, suggesting that combination therapy may provide superior suppression of neutrophil-mediated inflammation (Figure 3).

EN-RAGE levels were higher in SpA patients than in controls and further increased with disease severity, reinforcing its value as an inflammatory marker in SpA [17]. Additionally, patients receiving NSAID therapy had higher EN-RAGE levels than those on TNF inhibitors, indicating that TNF blockade may more effectively reduce EN-RAGE expression, likely through the modulation of innate immune pathways (Figure 2).

IL-17 levels, by contrast, were not significantly different between SpA subgroups and the healthy controls. This is consistent with previous studies that have demonstrated only weak or no correlations with clinical disease activity indices such as the Bath Ankylosing Spondylitis Disease Activity Index (BASDAI), Bath Ankylosing Spondylitis Functional Index (BASFI), or Axial Spondyloarthritis Disease Activity Score (ASDAS) [30,31,32,33]. This limited association can be explained by several factors. Biologically, IL-17 is predominantly produced and acts locally at sites of inflammation (entheses, synovium, skin, gut), and systemic measurements may not fully capture local cytokine activity. In addition, disease activity is mediated by multiple inflammatory pathways, with fluctuating dominance of different cytokines, and serum IL-17 levels have a short half-life with rapid dynamic changes. Methodologically, patient-reported outcomes (e.g., BASDAI) may not directly reflect inflammatory burden, and there may be a temporal lag between cytokine changes and clinical manifestations. Clinically, non-inflammatory pain sources or comorbidities can further weaken the correlation. Thus, IL-17 is unlikely to be a reliable serum biomarker for disease monitoring, despite its clear pathogenic relevance in SpA. However, downstream molecules induced by IL-17 (e.g., alarmins, defensins) may provide more robust systemic indicators of disease activity [21].

A limitation with regards to the measurement sensitivity of IL-17 is that the concentration of this cytokine was below the level of detection of the kit used in several of our samples. Nevertheless, these low concentrations could also be a reflection of the fact that IL-17 is often involved in local inflammatory processes at the tissue level, and its systemic concentrations, produced in peripheral blood, may be significantly lower compared to tissue levels.

Upon SA1 stimulation, we observed significant increases in TNF-α, HNP-1–3, and calprotectin levels across all patient subgroups, reflecting the activation of proinflammatory pathways. Notably, these effects were attenuated by co-treatment with SZR72, confirming the anti-inflammatory potential of this KYNA analog. The modulatory effect of SZR72 was evident even in untreated healthy samples, suggesting a broad spectrum of immune regulation [9].

Unexpectedly, patients with high disease activity showed lower basal TNF-α levels. This may reflect enhanced clearance via soluble TNF receptors, a cytokine shift toward IL-6/IL-17 pathways, or compartmentalized cytokine production at sites of inflammation. In addition, chronic immune activation could induce a state of cellular exhaustion, limiting basal TNF-α release.

Consistent with our previous studies, TSG-6 levels increased upon SA1 stimulation, and were further augmented by SZR72 in most SpA patient groups, further indicating the anti-inflammatory effects of this compound by enhancing the production of TSG-6 [8,9]. However, in patients receiving NSAID or csDMARD monotherapy, the additive effect of SZR72 was diminished. This could imply that these conventional therapies already partially engage the TSG-6 pathway, limiting the benefit of further pharmacological upregulation. These findings raise the possibility that TSG-6-targeted interventions may be most effective in therapy-naïve or bDMARD-treated populations.

S100A12 showed only a mild response to SA1 or SZR72, with no significant changes in patients with axial or peripheral SpA, those on NSAID or DMARD therapy, or in patients with severe disease. This may indicate that S100A12 levels are less modifiable by KYNA-based interventions in SpA or that expression is already saturated in active disease states.

As expected, IL-17 levels remained unchanged following SA1 and SZR72 treatment across all groups. This supports the hypothesis that IL-17 acts predominantly in the local tissue environment and is minimally represented in peripheral blood [20]. However, since IL-17 drives the expression of several downstream proinflammatory mediators (including those modulated by SZR72), KYNA analogs may exert their therapeutic effects by targeting these secondary pathways, rather than IL-17 directly.

Our data also suggest that peripheral immune modulation with KYNA analogs may indirectly reflect or even influence inflammation in target tissues, such as the joints or extra-articular organs. Further studies are warranted to assess the tissue penetration and systemic distribution of SZR72, including its potential to cross the blood–synovial barrier.

In summary, our results provide new evidence that the KYNA analog SZR72 can suppress key proinflammatory mediators (TNF-α, HNP-1–3, calprotectin) and enhance anti-inflammatory TSG-6 expression in SpA patients. The findings also highlight that certain biomarkers (e.g., EN-RAGE, IL-17) are less responsive to systemic modulation, likely due to their localized expression or intrinsic resistance to specific immune interventions. These insights support further exploration of kynurenine pathway modulators as potential adjunctive therapies in SpA.

## 4. Materials and Methods

### 4.1. Patients

AS patients were classified according to the ASAS classification criteria for axial (*n* = 43) [1,34] and peripheral (*n* = 16) [1] SpA and PsA patients (*n* = 38) were classified according to the CASPAR classification criteria [35]. The patient characteristics and clinical data are presented in Table 2. Patients with SpA (*n* = 97) were further grouped based on peripheral disease activity score in 28 joints (DAS28) of ≤2.6, 2.6 ≤ 3.2, 3.2 ≤ 5.1, and ≥5.1—remission, mild, moderate, and severe, respectively. The axial involvement was defined with the BASDAI activity score. The cut-offs selected to separate these states were as follows: <2.8—inactive/mild disease; 2.8 ≤ 4: moderate disease; and >4: high disease activity [36]. AS patients had either axial (*n* = 43) or axial + peripheral (*n* = 16) activity, while all PsA patients had peripheral (*n* = 38) involvement but in approximately half of the patients axial (*n* = 19) activity was also present.

Patients with SpA were treated with anti-TNF-α monotherapy (*n* = 65), conventional synthetic disease-modifying antirheumatic drug (csDMARD) monotherapy (*n* = 9) including methotrexate (*n* = 6), leflunomide (*n* = 2), sulfosalazine (*n* = 2), anti-TNF-α combined with DMARDs (*n* = 20) including methotrexate (*n* = 12), leflunomide (*n* = 4), sulfasalazine (*n* = 4), IL-17 inhibitor (*n* = 2), NSAID monotherapy (*n* = 7), and steroid monotherapy (*n* = 3). HLA-B27 analysis was carried out with flow cytometric immunophenotyping method. As a control group, 11 healthy volunteers were recruited. These healthy individuals had a negative history of arthritis and negative status based on laboratory and physical examinations. This study was executed with the approval of the Ethics Committee of the University of Szeged (ETT-TUKEB 149/2019-SZOTE) and in full accordance with the Declaration of Helsinki (1964).

### 4.2. KYNA Analog SZR 72

In our experiments, a novel KYNA analog, N-(2-N, N-dimethylaminoethyl)-4-oxo-1H-quinoline-2-carboxamide hydrochloride (SZR72), was used. It was designed and synthetized in the Institute of Pharmaceutical Chemistry and Research Group for Stereochemistry, Hungarian Academy of Sciences, University of Szeged. The synthesis is carried out with the coupling of KYNA and 2- dimethylaminoethylamine and, later, treatment with ethanolic hydrogen chloride, resulting in the above-mentioned SZR72 KYNA analog. The unique structure of SZR72 allows it to cross the blood–brain barrier as its membrane permeability increased due to the presence of water-soluble side chains with an extra cationic center [11,37,38].

This KYNA analog was dissolved in phosphate-buffered saline [37]. In our previous experiments, the inhibitory effects of kynurenic acid and a kynurenic acid analog SZR 72 were investigated, and 25 μM SZR72 proved to be ineffective. At increasing concentrations (125, 250, and 500 μM), SZR72 exhibited increasing inhibitory effects on TNF-α production. Therefore, similar to our previous work in rheumatoid arthritis, only the result with the most effective concentration (500 μM) is demonstrated in this paper [9,12].

### 4.3. Human Blood Incubation Method

Ethylenediaminetetraacetic acid (EDTA)-anticoagulated peripheral venous blood samples from 38 PsA, 54 AS patients, and 11 healthy controls were taken. Subsequently, 1 mL each of the blood samples were incubated for 18 h in a Heracell CO_2_ incubator (Thermo Fischer Scientific, Waltham, MA, USA) at 37 °C, with and without the presence of 10^7^/mL heat-inactivated *Staphylococcus aureus* (SA1). SA1 was used as a TNFα and inflammatory cytokine inducer [39]. In parallel experiments, blood samples were pretreated with SZR72 at 500 µM concentration for 30 min before the induction with SA1. The KYNA analog was dissolved in phosphate-buffered saline (PBS), then diluted in Roswell Park Memorial Institute (RPMI) medium (SIGMA). Thereafter, 100 µL of this mixture was added to the blood samples, while 100 µL of RPMI was added to all the other blood samples without SZR72 to balance the volumes. After incubation, the samples were centrifuged at 3000× *g*, after which the supernatants were obtained and tested by enzyme-linked immunosorbent assay (ELISA) for TNF-α (SIGMA, St. Louis, MO, USA, detection range 15.6–1000 pg/mL; with a two-times sample dilution), TSG-6 (Fine Biotech, Wuhan, China, detection range 0.05–100 ng/mL; with a two-times sample dilution), S100A8/9 (calprotectin) (Hycult-Biotech, HK373-02, Uden, The Netherlands, detection range 15.6–1000 pg/mL, with a fifty-times sample dilution), S100A12 (EN-RAGE) (CircuLex CY-8058 V2, MBL International Corporation, Woburn, MA, USA, detection range 15.6–1000 pg/mL with a two-hundred-times sample solution), HNP1–3 (Hycult-Biotech HK324, Uden, The Netherlands, detection range 0.625–40 ng/mL, with a two-times sample dilution), and IL-17 (Fine Biotech, Wuhan, China, detection range 15.6–1000 pg/mL, with a two-times sample dilution) [9]. The same donor sample was used for all cytokine assays. Each concentration was tested in duplicate.

### 4.4. Statistics

The Kruskal–Wallis test was used to compare different patient groups and healthy controls. To compare the concentrations of mediators within a group of patients, the Friedman test with Dunn’s post-test was used, and *p* < 0.05 was considered significant. The Graph-Pad Prism 10 statistical program (Graph Pad Software Inc., San Diego, CA, USA) was used for the calculations.

## Figures and Tables

**Table 1:** Data are expressed as median [interquartile range]. * *p* < 0.05 vs. untreated, ** *p* < 0.01 vs. untreated, **** *p* < 0.0001 vs. untreated, ^#^ *p* < 0.05 vs. +SA1, ^##^ *p* < 0.01 vs. +SA1, and ^####^ *p* < 0.0001 vs. +SA1. TNF-α: tumor necrosis factor-α; TSG-6: tumor necrosis factor-stimulated gene-6 protein, EN-RAGE: extracellular newly identified receptor for advanced glycation end-products-binding protein; HNP1–3: human neutrophil peptide 1-3; IL-17: interleukine-17, SpA: spondyloarthritis, SA1: heat-inactivated *S. aureus*, SZR72: kynurenic acid analog, and BLD: below level of detection.

	Controls *n* = 11	SpA Remission/Mild *n* = 65	SpA Moderate *n* = 20	SpA Severe *n* = 12
TNF-α pg/L	8.9 [BLD-350]	73 [BLD-1122]	79 [BLD-900]	43 [BLD-1479]
TNF-α pg/mL + SA1	444 [140–1030] ****	280 [BLD-1800] ****	333 [60–1800] ****	339 [68–1945] ****
TNF-α pg/mL + SA1 + SZR72	280 [60–838] ** ##	131 [BLD-1180] ####	145 [BLD-1200] * ##	199 [39–1643] #
TNF-α + SA1 fold change	19.2 [10–48.7] ****	2.4 [1.4–6.5] ****	3.6 [2.6–10.7] ****	8.7 [2.1–17.1] ****
TNF-α + SA1 + SZR72 fold change	8.3 [4.3–26.0] ** ##	1.2 [1.0–3.0] ####	2.0 [1.3–6.0] * ##	5.2 [1.1–17.3] #
TSG-6 ng/ml	4.99 [0.1–20.12]	2.46 [0.05–18]	3.76 [0.08–25.23]	0.8 [0.001–4.25]
TSG-6 ng/mL + SA1	9.22 [2–24] **	4.81 [0.51–30] ****	6.2 [0.31–28.3] **	4.25 [0.09–20.58] *
TSG-6 ng/mL + SA1 + SZR72	13.6 [6.7–44] **** ##	8.34 [2.3–37.5] **** ####	9.78 [2.31–35.6] **** ##	6.94 [0.25–26.29] ****
calprotectin ng/ml	824 [390–1902]	849 [55–2500]	865.88 [442–2618]	3733 [590–14,770]
calprotectin ng/mL + SA1	12,350 [4726–15,500] ****	8582 [2808–18,800] ****	9313 [5110–21,280] ****	25,950 [5685–41,203] ****
calprotectin ng/mL + SA1 + SZR72	5892 [1020–15,490] ** ##	5806 [75–15,567] **** ####	7010 [4000–19,500] ** #	22,250 [384–36,211] #
calprotectin + SA1 fold change	13.9 [8.0–15.0] ****	9.6 [7.9–14.0] ****	9.8 [7.5–12.7] ****	6.6 [5.1–8.0] ****
calprotectin + SA1 + SZR72 fold change	7.7 [4.0–10.5] ** ##	7.2 [5.3–11.0] **** ####	7.5 [5.7–11.2] ** #	4.9 [3.5–6.6] #
EN-RAGE ng/mL	8306 [1150–12,000]	11,864 [1066–24,738]	12,500 [7114–52,766]	19,288 [1560–93,742]
EN-RAGE ng/mL + SA1	13,098 [10,074–16,955] ****	13,659 [2655–88,000] ****	14,416 [4588–53,967] **	19,678 [9536–133,215]
EN-RAGE ng/mL + SA1 + SZR72	12,932 [9744–14,129] **	12,440 [2357–24,503] ****	13,136 [6433–48,536]	21,391 [10,100–109,101]
HNP1–3 ng/ml	121 [32–986]	98 [16–1622]	119 [30–3000]	1828 [62–12,950]
HNP1–3 ng/mL + SA1	540 [71–1500] ****	520 [32–13,000] ****	535 [200–3025] ****	6345 [176–51,300] ****
HNP1–3 ng/mL + SA1 + SZR72	387 [30–1270] * #	280 [17–11,000] **** ####	272 [110–2800] ##	4850 [150–36,000]
IL-17 pg/ml	184 (BLD-4000)	BLD (BLD-4000)	88.9 (BLD-4000)	79.9 (BLD-4000)
IL-17 pg/mL +SA1	189.5 (BLD-3773)	BLD (BLD-4000)	326.9 (BLD-4000)	216.3 (BLD-4000)
IL-17 pg/mL +SA1 + SZR72	BLD (BLD-1736.5)	BLD (BLD-4000)	301.69 (BLD-4000)	132.95 (BLD-4000)

**Table 2:** Clinical characteristics of healthy individuals and SpA patients. Data are expressed as median [interquartile range]. SpA: spondyloarthritis, HLA-B27: human leukocyte antigen B27, CRP: C-reactive protein, and ESR: erythrocyte sedimentation rate.

Characteristics	Healthy Controls *n* = 11	SpA Remission/Mild *n* = 65	SpA Moderate *n* = 20	SpA Severe *n* = 12
Age (years)	46 (25–52)	49 (24–81)	46 (18–75)	54 (34–72)
Gender (male/female)	4/7	33/27	12/8	7/5
Duration of disease (years)	-	15 (1–49)	9 (0–29)	8 (0–19)
HLA-B27 (positive/negative/no data)	-	35/15/15	7/8/5	6/2/4
CRP (mg/L)	-	4.55 (0.4–20)	18 (1–38)	36 (10–116)
ESR (mm/h)	-	9 (1–65)	32 (1–99)	49 (13–120)

## Data Availability

The original contributions presented in this study are included in the article. Further inquiries can be directed to the corresponding author.
